# Integrated digital system for community engagement and community-based surveillance during the 2014–2016 Ebola outbreak in Sierra Leone: lessons for future health emergencies

**DOI:** 10.1136/bmjgh-2020-003936

**Published:** 2020-12-21

**Authors:** Mohamed F Jalloh, Paul Sengeh, Nyuma James, Saiku Bah, Mohammad B Jalloh, Katharine Owen, Samuel Abu Pratt, Allan Oniba, Musa Sangarie, Samuel Sesay, Jamie Bedson

**Affiliations:** 1 Department of Global Public Health, Karolinska Institutet, Stockholm, Sweden; 2 Focus 1000, Freetown, Sierra Leone; 3 Restless Development Sierra Leone, Freetown, Sierra Leone; 4 GOAL, Freetown, Sierra Leone; 5 BBC Media Action, Freetown, Sierra Leone; 6 Health Education Division, Sierra Leone Ministry of Health and Sanitation, Freetown, Western Area, Sierra Leone

**Keywords:** viral haemorrhagic fevers, public health

## Abstract

Community engagement and community-based surveillance are essential components of responding to infectious disease outbreaks, but real-time data reporting remains a challenge. In the 2014–2016 Ebola outbreak in Sierra Leone, the Social Mobilisation Action Consortium was formed to scale-up structured, data-driven community engagement. The consortium became operational across all 14 districts and supported an expansive network of 2500 community mobilisers, 6000 faith leaders and 42 partner radio stations. The benefit of a more agile digital reporting system became apparent within few months of implementing paper-based reporting given the need to rapidly use the data to inform the fast-evolving epidemic. In this paper, we aim to document the design, deployment and implementation of a digital reporting system used in six high transmission districts. We highlight lessons learnt from our experience in scaling up the digital reporting system during an unprecedented public health crisis. The lessons learnt from our experience in Sierra Leone have important implications for designing and implementing similar digital reporting systems for community engagement and community-based surveillance during public health emergencies.

**Summary box**

- Large-scale, localised community engagement was necessary to directly involve communities in the Ebola response and rapidly change traditional burial practices and health-seeking behaviour. Community engagement and community-based surveillance were digitally integrated and scaled-up in six high transmission districts in Sierra Leone.
- Paper-based reporting posed considerable logistical challenges in monitoring community engagement activities, getting community feedback, and reporting suspected cases and deaths in communities.
- The experience of implementing the digital reporting system shed light on the following lessons: (1) technological tools should be driven by the real data needs for epidemic control; (2) the people and users of the technology should be put at the centre and not be overshowed by the technology; (3) instead of trying to design a perfect system, it is more useful to build by doing so that you can turn your challenges into learning opportunities; (4) data collection should be integrated as a key ingredient of structured community engagement (not as a standalone activity), and it should complement and inform interaction between communities and trusted interlocutors; (5) increased financial investments are needed to maximise the benefit of a digital reporting system to translate community-based data into action; (6) data reporting synergies are possible at the community level despite missed opportunities for formal data integration.
- The lessons we learnt in transitioning to a digital data collection and reporting system for community engagement and community-based surveillance have implications for responding to other health emergencies.

## Background

The 2014–2016 epidemic of Ebola virus disease (Ebola) in Sierra Leone, Liberia and Guinea remains the largest documented outbreak of Ebola to date.^1^ The epidemic was fuelled by unsafe traditional burials that involved physical contact with corpses and delays in medical care-seeking behaviours that were partly due to the social stigma of Ebola, fear and community distrust in the response.^2–4^ Building and sustain trusting with communities was necessary to rapidly change traditional burial practices and health-seeking behaviours.^5–9^

In Sierra Leone, the social mobilisation pillar was established in June 2014 to coordinate and provide strategic oversight of community engagement activities across all 14 districts.^10^ The Social Mobilisation Action Consortium (SMAC) was formed in September 2014 to support the pillar in scaling-up structured, data-driven community engagement.^11^ As part of the consortium, GOAL Ireland and Restless Development recruited, trained and supported 2500 community mobilisers to implement Community-led Ebola Action planning.^12^ FOCUS 1000, a local non-governmental organisation, engaged almost 6000 religious leaders to promote Ebola prevention practices, especially safe burial measures.^13^ BBC Media Action supported 42 local radio stations to improve the quality of Ebola risk communication through various radio programmes.^14^

## Cross-cutting challenges with paper-based reporting

Digital data collection was not common in Sierra Leone prior to the Ebola outbreak. National surveys and census were done using paper-based means.^15 16^ Cross-cutting logistical challenges with paper-based reporting during the response included time spent on verifying and understanding the handwritten forms as well as additional time for data entry, cleaning and processing.^17^ Although paper-based reporting tools were initially used by community mobilisers to a considerable degree of success, over time, cross-cutting challenges were also experienced in deploying the paper-based tools across all partner organisations.^11^

## Intervention-specific challenges with paper-based reportingning from paper-based to digital data

Establishing a functional paper-based reporting for religious leaders would have required a separate category of paid religious leaders or additional SMAC staff to physically travel to collect the weekly reporting forms. Another alternative was to provide the trained religious leaders with transportation allowance to travel to a central location on a weekly basis to submit their reports to SMAC staff. Both options would have significantly increased the cost of the religious leader intervention, added time to obtain the reports, and posed additional data quality issues. SMAC partner radio stations also initially lacked a functional way to systematically report their weekly radio programming activities.

## Transitioning from paper-based to digital data collection

In January 2015, SMAC received a grant from the Bill & Melinda Gates Foundation to transition from paper-based to digital reporting in six prioritised high transmission districts (Kambia, Kono, Moyamba, Port Loko, Western Area Rural, Western Area Urban).^18^ The timeline of the system’s rapid design and deployment is outlined in table 1. The consortium established a Monitoring and Evaluation Working Group to oversee the deployment of the Digital Data Collection System (DDCS). The Working Group together with 10 fulltime staff trained a group of 30 principal trainers using a standard curriculum (online supplemental material). By April 2015, there were 1400 phones registered on the DDCS and 2800 persons trained on the system (table 2). The number of actively registered phones on the DDCS fluctuated over time and reached up to 1500 by June 2015.

10.1136/bmjgh-2020-003936.supp1 Supplementary data

10.1136/bmjgh-2020-003936.supp2 Supplementary data

**Table 1:** Timeline of outputs for deploying the digital reporting system, Social Mobilisation Action Consortium (SMAC), Sierra Leone, January–March 2015

Outputs	Timelines (2015)	
	Planned	Actual
Project management and information technology infrastructure established to oversee rapid data collection, action triggering, and repository building	15 January	10 February
Data management staff recruited and trained	31 January	10 February
Data analysis and reporting plan finalised	31 January	10 February
ODK and SMS data collection platforms customised and tested	2 February	10 February
Training materials developed and finalised	4 February	10 February
DDCS deployed in Western Area Urban and Western Area Rural districts where Ebola transmission was highest at the time	15 February	31 March
DDCS deployed in remaining four high transmission districts	22 February	21 March
SMAC community-based EVD surveillance and reporting initiated and operational in all six high transmission districts	28 February	23 March

DDCS, Digital Data Collection System; EVD, Ebola virus disease; ODK, Open Data Kit; SMS, short message service.

**Table 2:** Distribution of formally trained community reporters and registered phones on the Social Mobilisation Action Consortium’s digital data collection system by April 2015

	GOAL Ireland	Restless development	FOCUS 1000	BBC media action	Total
# of community mobilisers trained	1038	526	n/a	n/a	1564
# of religious leaders trained	n/a	n/a	1016	n/a	1016
# of radio station managers trained	n/a	n/a	n/a	202	202
*Total # of trained community reporters*	1038	*526*	1016	*202*	2800
*Total # of phones registered on DDCS*	*519*	*263*	*508*	*110*	1400

#, number; DDCS, Digital Data Collection System; n/a, not applicable.

## System description

**Figure 1:**
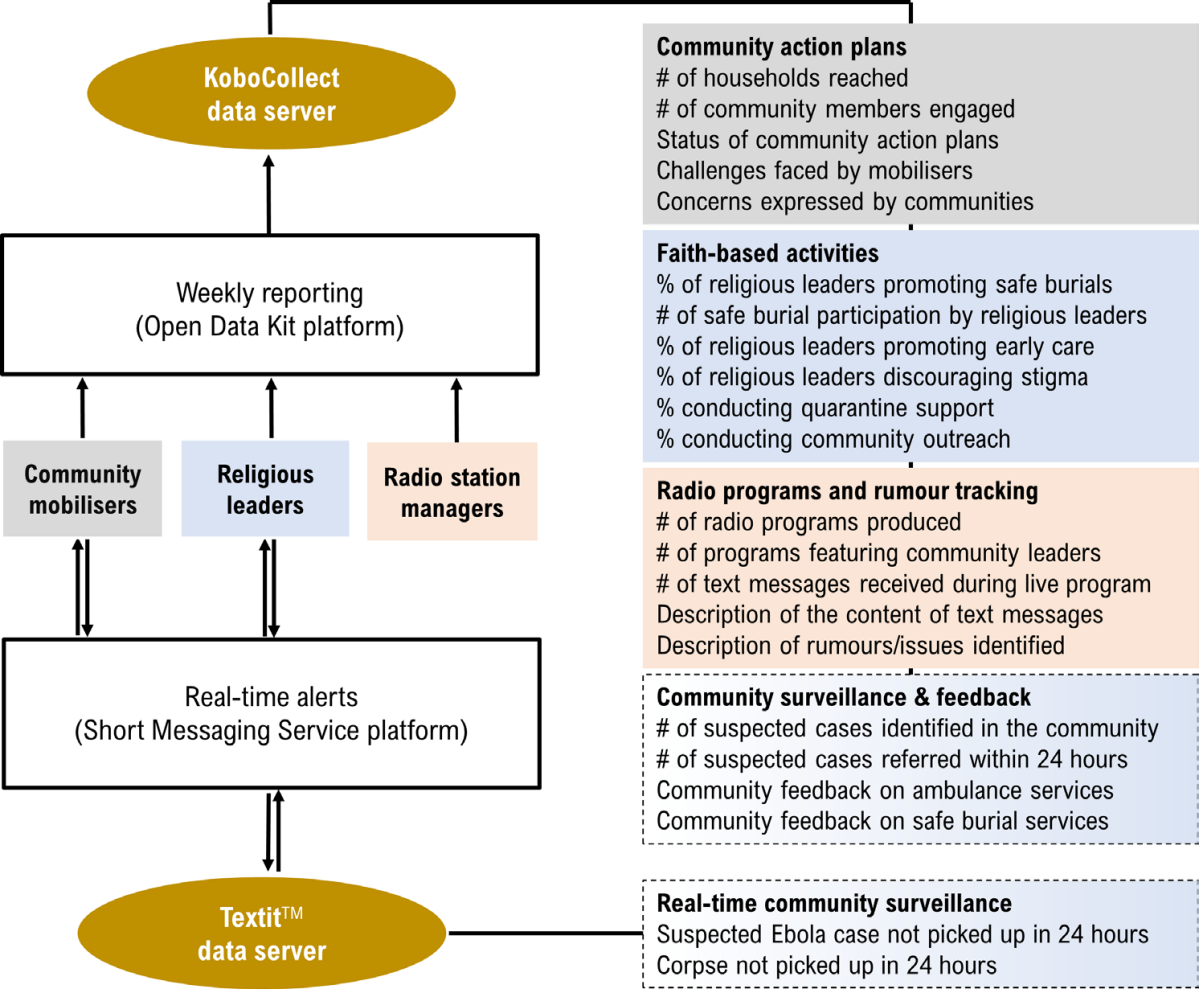
Conceptual framework for the Social Mobilisation Action Consortium (SMAC) ’s digital data collection system during the 2014–2016 Ebola outbreak in Sierra Leone.

The DDCS comprised daily and weekly reporting mechanisms using Android-based smart phones (figure 1). Daily reporting consisted of using an interactive short message service (SMS) to send real-time text message alerts of sick people that needed ambulance services and deaths in the community that required safe burials. The Textit platform (Rwanda: Nyaruka and UNICEF) was used for the real-time SMS reporting.^19^ SMS alerts were channelled to liaison officers that were integrated into the district-level response. The liaison officers confirmed further reported information before connecting with the district-based burial and surveillance teams to facilitate prompt action.

Weekly reporting consisted of data reporting components on community engagement activities, community feedback and community-based surveillance of sick people and deaths. The Open Data Kit (ODK) platform (www.opendatakit.org)^20^ was used for the weekly reporting. The ODK form was based on an established list of closed-ended and open-ended items initially used by community mobilisers and eventually expanded for use by religious leaders and radio stations.^21^

## Summary and interpretation of the reported data

**Figure 2:**
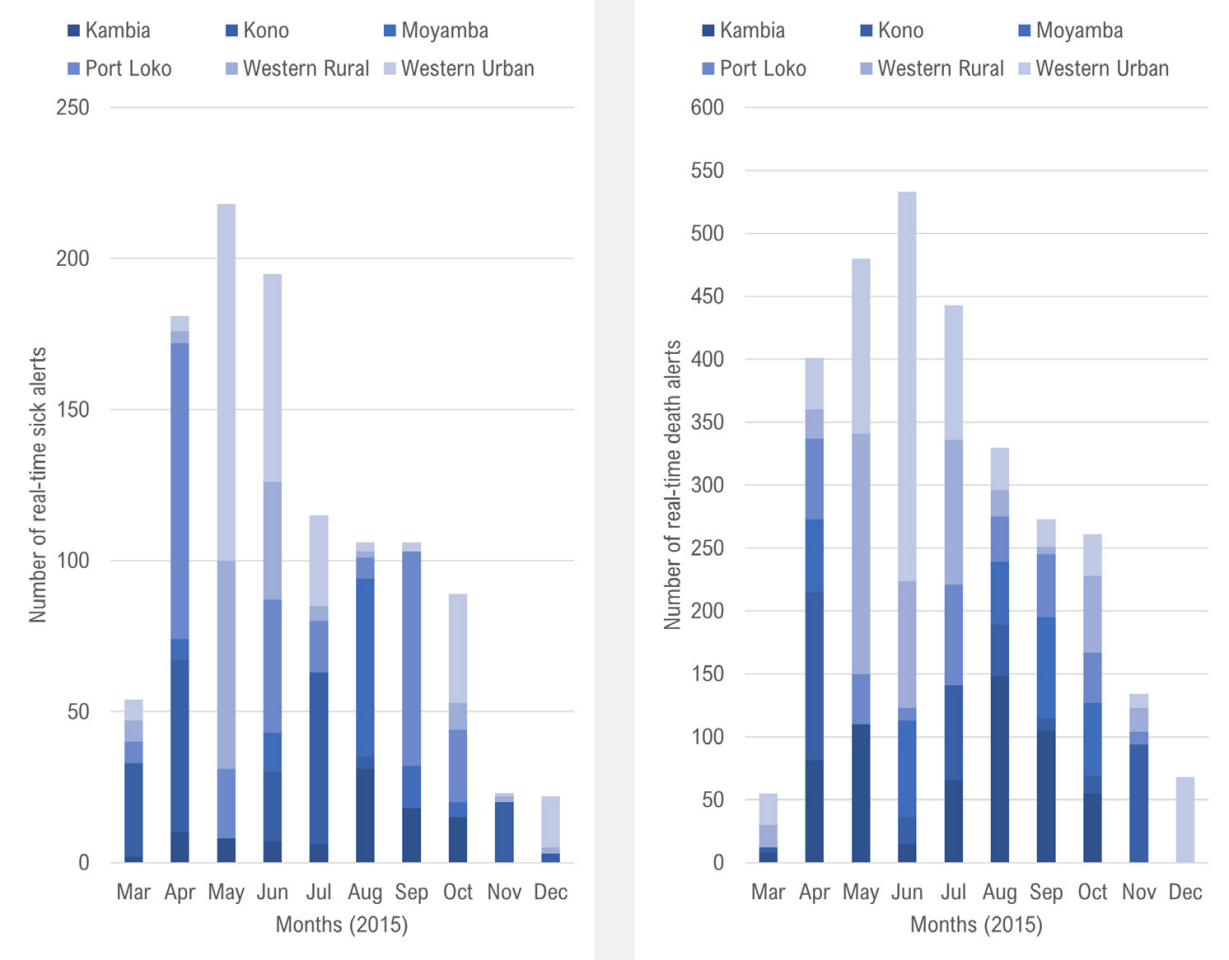
Number of daily real-time alerts on suspected Ebola patients and deaths needing response services submitted through the Textit platform, Social Mobilisation Action Consortium, Sierra Leone, March–December 2015.

**Figure 3:**
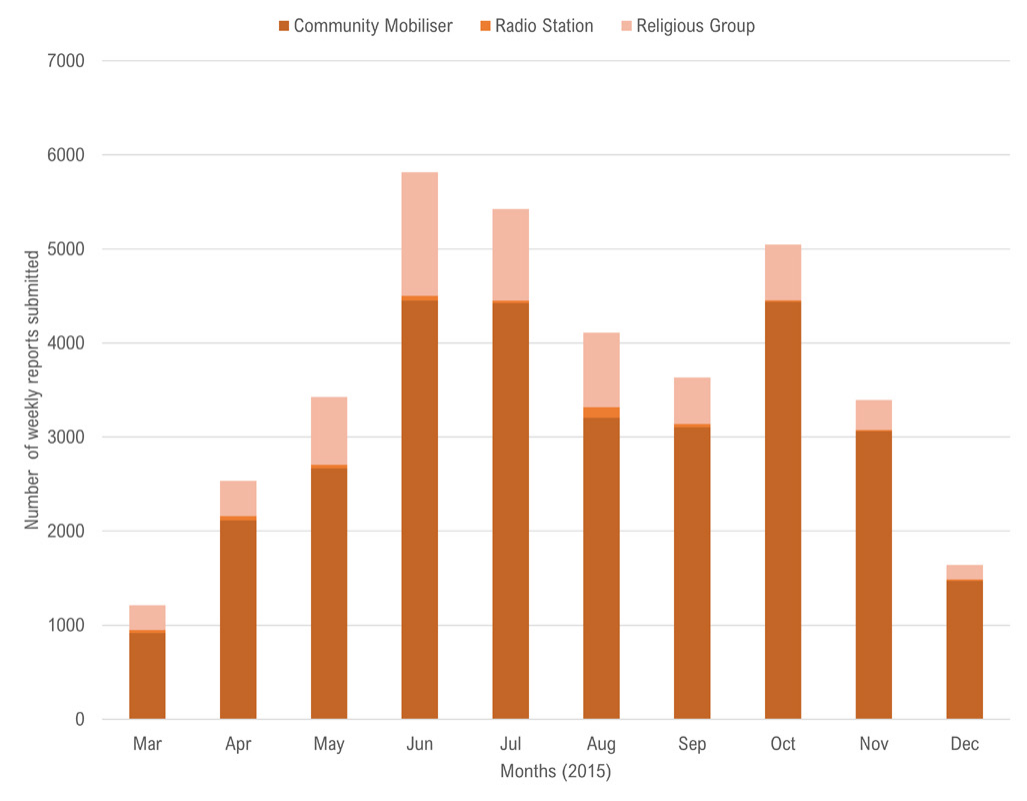
Number of community engagement reports submitted weekly through Open Data Kit platform, Social Mobilisation Action Consortium, Sierra Leone, March–December 2015.

Between 30 March and 31 December 2015: 1110 alerts of sick people that needed ambulance services and 2978 alerts of deaths that needed safe burial services were reported in real-time through the SMS platform (figure 2). During this period, the number of alerts submitted regarding sick people was highest in May 2015 and the number of death alerts were highest in June 2015. Between 26 March and 31 December 2015: 36 619 weekly reports on activities and community feedback were submitted via the ODK platform (figure 3). The number of weekly reports submitted was highest in June 2015 (n=5818), which represented 97% of the expected 6000 monthly total.

Starting in July 2015, the submitted ODK reports and SMS alerts steadily declined until December 2015, which was largely due to lower level of reporting by community mobilisers, religious leaders and radio station managers. The decline in reported alerts should not be necessarily interpreted as commensurate with the decline in the actual numbers of suspected cases and deaths in communities. Nevertheless, the overall waning of the epidemic during this period and the declaration of the end of the epidemic in November 2015 may have influenced the reduced level of reporting as volunteers likely became less vigilant in their reporting.

A full cycle of digital reporting via ODK took 10–12 days on average (compared with 12–15 days on average using paper-based tools by mobilisers). The bulk of the time (7 days on average) was spent on the community engagement activities and simultaneous reporting of the data at the end of the week; followed by 3–5 days for data processing and analysis (compared with up to 8 days using paper-based tools). On the other hand, SMS alerts of suspected cases and deaths in the community were transmitted in real-time to liaison officers who usually followed up to gather additional information on the alert within the same day and shared the available alert details with district-based surveillance officers. The merged data from the SMAC intervention have been publicly released^21^ and used in secondary analysis to understand the social and behavioural dynamics of the Ebola epidemic.^22^

## What did we learn?

### 1. Technological tools should be driven by the real data needs for epidemic control

We ensured that the technology did not drive the process. Instead, the identified *data needs and context* steered the decisions made about the technological platforms that were leveraged to address our priorities. We aligned the strengths of the technological platforms with the data needed to inform the response. For example, Textit was suitable for sending the alerts on sick people and deaths in communities because it was SMS-based and allowed real-time interactive reporting. The direct Short Message Peer-to-Peer (SMPP) connection with the telecommunication company was important to enable large volumes of SMS. For the weekly reporting, we needed a platform that could handle the large quantum of data that the SMAC was collecting to monitor community engagement activities and community surveillance of sick people and deaths to gain insights into important trends over time as the outbreak evolved. A cross-cutting dimension to having the needs drive the process is making a careful determination on the type of data to be collected as well as the scope and frequency of data collection. We learnt that it is important to prioritise the *required data* and avoid collecting information that may be *good to have but not necessary*.

### 2. Put people at the centre when introducing new technology

We needed to tailor the digital tools appropriately to our target users. This was partly accomplished by holding user-testing sessions that provided feedback on the system’s design for low-literacy users. We ensured that the questions/items in the SMS and ODK platforms were in simple-to-understand language. A dedicated short code number (334) was used to transmit all text messages for ease of reporting. Feedback on the DDCS were regularly solicited through telephone discussions and SMS exchanges between trained community reporters and the data management staff. For instance, in the first 2 months of deploying the system, users reported problem about the irregularity of getting telecommunication connectivity to allow sending of the ODK reports and SMS failures due to disruptions in the SMPP connection. In responding to this feedback, we conducted a troubleshooting activity in May 2015. Our team developed a troubleshooting form to preidentify the range of issues in each district. We recurrently observed that the users changed their configuration settings in the phones, which prevented them from having data access to submit the ODK forms. A month later in June 2015, we then conducted refresher trainings. Both the troubleshooting visits in May and the refresher trainings in June increased reporting levels in those months compared to the prior months. While these large-scale, in-person follow-ups were costly to be done on a monthly basis, similar community-based data reporting efforts should sufficiently budget and plan for monthly follow-up with users to optimise real-time data utilisation.

### 3. Do not wait for a perfect system and instead build by doing

As the system was being rolled out in March 2015, the Government of Sierra Leone instituted a 3-day stay-at-home campaign. Trained community mobilisers and religious leaders teamed up with outbreak response teams to conduct house-to-house visits to educate households about Ebola and to actively identify cases during the campaign. The SMS component of the DDCS was rapidly customised to report real-time community feedback on the campaign to the response leadership (online supplemental material). Misinformation and concerns identified were addressed through interactive radio programming and subsequent household visits during the campaign.

Since we lacked experience using the Texit platform, we quickly learnt from UNICEF’s use of RapidPro for its UReport, which was built on the same SMS platform as Texit. We learnt from UNICEF about the potential roadblocks when integrating the SMS platform with the telecommunication service providers. With that in mind, we spent a significant amount of time upfront to ensure that we had a stable SMPP integration with the telecommunication providers. Despite efforts to anticipate and plan for this challenge, we ultimately experienced recurring SMPP connection interruptions that sometimes prohibited our users from submitting SMS alerts. We eventually had to migrate to a different telecommunication service provider that was able to provide a more stable connection. We first observed how efficiently the new provider handled the SMPP integration with the Texit platform before deciding on a full migration. While it took nearly 2 months for the first provider to execute the SMPP connection, the second provider did so in just 2 days. We started the migration with 100 users in Western Area to first learn from the experience and then continued with a phased migration of all users.

### 4. Data collection by community mobilisers and leaders is not a standalone activity

Data were collected by community mobilisers, religious leaders and radio station managers as an integrated part of their community engagement roles.^12^ Data collection was not an end in itself. Our primary focus was to understand when and how community mobilisers and religious leaders engaged with local communities. We then carefully integrated key elements of community surveillance with community feedback. The approach aimed to avoid the idea of ‘listening in’ to communities for the purposes of data collection alone, but of ‘listening to’ communities whereby the data informed a two-way communication process. Putting digital technology in the hands of community mobilisers and religious leaders to undertake grassroots community surveillance has the advantage of using established community structures and trusted interlocutors to collect data to inform the response. Leveraging community assets and resources for public health surveillance holds tremendous value in responding to future health emergencies, especially when surveillance efforts can be integrated into sustained community engagement.

### 5. Increased financial investments are needed to translate community-based data into action

Nearly US$600 000 was spent on the digital reporting system between February and December 2015. Start-up costs for procuring the phones plus recurring costs for Internet connectivity and SMS charges comprised the largest expense category followed by personnel costs. Excluding start-up costs, the all-inclusive, monthly running cost was approximately US$32 000 on average between February and December 2015. With the continuous reduction in the market cost of basic smart phones and decreased costs associated with mobile Internet connectivity, it is possible that future implementation of a similar system will incur lower start-up costs.

While a formal cost-benefit analysis is outside the scope of this paper, we should note that it is generally difficult to quantify the benefit of such system in financial terms alone. For instance, the more than 4000 real-time alerts of sick people needing ambulances to isolation centres and deaths needing safe burials represent a qualitative benefit that likely prevented secondary Ebola infections in thousands of households and communities. To the best of our knowledge, the more than 37 000 ODK reports submitted through the system represent the single largest community-based data repository available from the Ebola response in Sierra Leone, Liberia and Guinea. Use of ODK for reporting cut the reporting cycle by 3 days on average, which led to timelier availability of information on emerging rumours, challenges, action plans and feedback from communities to inform ongoing response options. Moreover, the system contributed to data-driven decision-making within the consortium as well as in the consortium’s advocacy for community-based solutions in the national and sub-national response.

Increased financial investments would have helped to maximise on the benefits of the system including the hiring of dedicated staff focusing on data utilisation. We underestimated the resources needed to rapidly analyse the data and inform national and district level response actions. With the limited staff available, it was difficult to keep up with the quantum of alerts that were generated by the SMAC mobilisers, religious leaders and community members. Although we tried to maximise using the data for response actions through the existing district-based staff such as our district liaison officers, in hindsight, we needed a much larger team of staff to optimise the use of the data. Similar challenges have been documented in the Democratic Republic of Congo regarding the real-time use of sociobehavioural data to inform Ebola epidemic control.^23^ Senior personnel from the respective SMAC partner organisations used the collected data in their day-to-day advocacy and technical assistance efforts to the Government of Sierra Leone, including in their daily participation in the Incident Management meetings at the National Ebola Response Centre.

### 6. Data reporting synergies are possible at the community level despite missed opportunities for data integration

The SMAC’s work was not formally linked with the surveillance pillar structures for community event-based surveillance (CEBS), which was in a nascent stage during our DDCS implementation.^24 25^ However, at the community level, informal connections were made by SMAC mobilisers and religious leaders with CEBS community monitors. SMAC’s liaison officers frequently shared alerts that they received with district surveillance officers, which may have provided duplicate alerts of events reported by CEBS community monitors. Duplicate alerts for the same event may have helped to confirm the event and/or provide additional information that may not have been present in the initial alert received. In some instances, alerts were likely only received from SMAC mobilisers and religious leaders because they had geographic coverage in areas where CEBS was not operational. Looking into the future, the interactive SMS component of the digital system used by SMAC for real-time reporting of key community events may prove helpful in the scaling up of CEBS in Sierra Leone and other similar settings, especially in areas where Internet connectivity remains a challenge.

## Conclusions

Our digital platforms provided a practical framework for collecting, analysing and reporting data on community engagement and community-based surveillance during the Ebola outbreak in Sierra Leone. Demonstrating the utility of the combined data, including the paper-based data, gave community engagement stakeholders a valuable seat at the table in the Ebola response in Sierra Leone. Since our implementation of the digital system during the Ebola epidemic in Sierra Leone, there has been considerable advancements in community-based digital reporting in outbreak contexts.^26^

However, the lessons learnt from our experience in Sierra Leone and the underlying data could be further leveraged to inform ongoing and future health emergencies. Implementation of similar systems should make considerable provision for real-time data use by having dedicated teams focusing on data utilisation. Community-level data collection efforts during health emergencies need to anticipate and plan for not just how to speed up data collection through digitalisation, but also how to improve data quality and use the data to inform response strategies. Given the increasing recognition of the value of community-level data during health emergencies, coordination is required to ensure harmonisation and interoperability of community-based data systems. Emerging digital tools offer unique opportunities to catalyse the timely collection of community feedback and monitoring of community-level behaviours during health emergencies. These digital tools should not be standalone and must be integrated into well-planned community engagement that enable response stakeholders to listen to and partner with communities.

As learnt from the 2014–2016 Ebola epidemic and more recent health emergencies including the COVID-19 pandemic, there is a continued need for increased financial investments to scale-up and integrate community-based data collection, analysis and synthesis into timely strategies and actions for epidemic control.

## Data Availability

Data are available in a public, open access repository. https://doi.org/10.6084/m9.figshare.8247002.v1
