# Supplementary material for: Integrated digital system for community engagement and community-based surveillance during the 2014–2016 Ebola outbreak in Sierra Leone: lessons for future health emergencies
Source: BMJ Glob Health. 2020 Dec 21;5(12):e003936. doi: 10.1136/bmjgh-2020-003936 (PMC7757454; doi:10.1136/bmjgh-2020-003936)
Supplement: Supplementary data [file bmjgh-2020-003936supp001.pdf]

Zero Ebola Campaign

Rapid SMS Survey with SMAC Community Mobilisers, Religious Groups, and Radio Stations

March 27th – 29th 2015

Social Mobilisation Action Consortium

smac

Background

- Rapid SMS survey was conducted with SMAC Community Mobilisers, Religious Groups, and Radio Stations during the Zero Ebola Campaign

- Sample size
  - Day1 (n=322); Day2 (n=399); Day3 (n=454)

    - Most respondents are from the high transmission districts; except for SMAC partner radio station managers who are also outside of the high transmission districts

Percent distribution of respondents by district

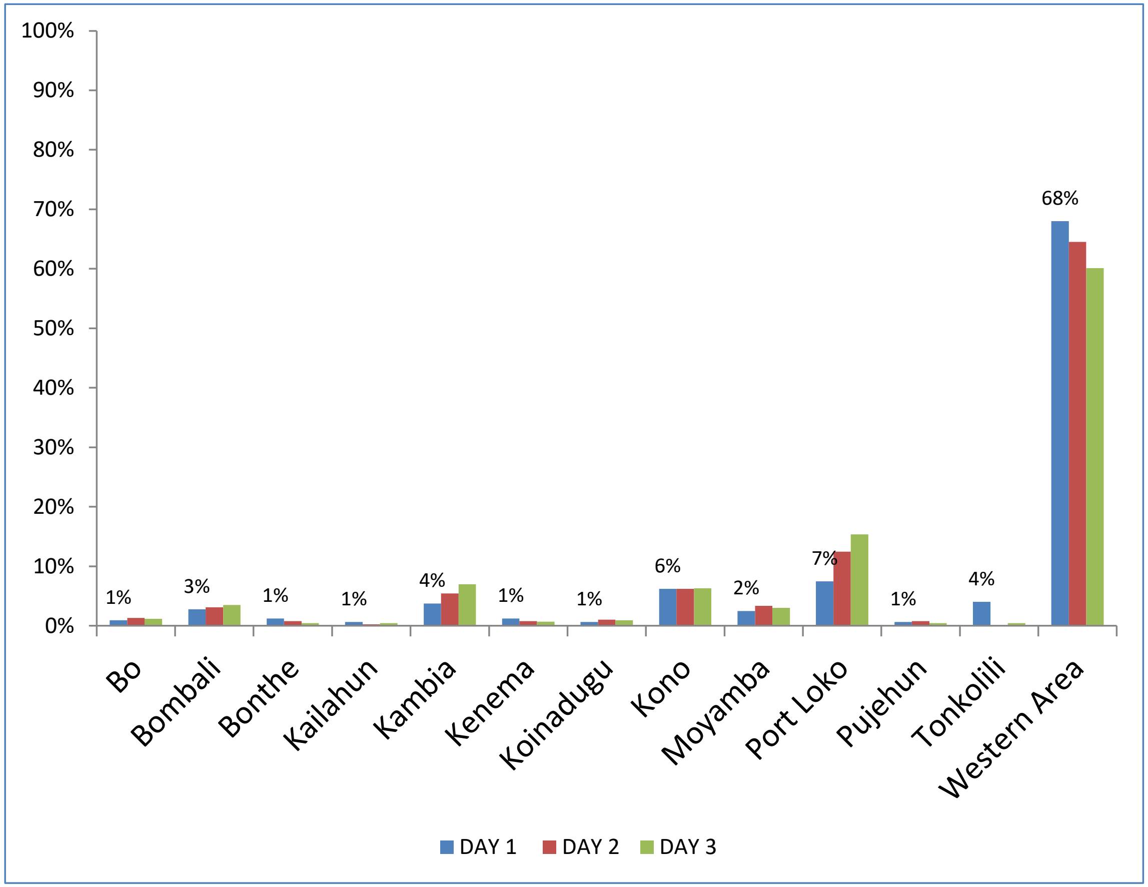

Note: The SMAC SMS system has not been fully rolled-out in all districts; Western Area is currently over-represented as the SMS system has been fully-rolled out in W.Area

Percent distribution of respondents by SMAC partner category

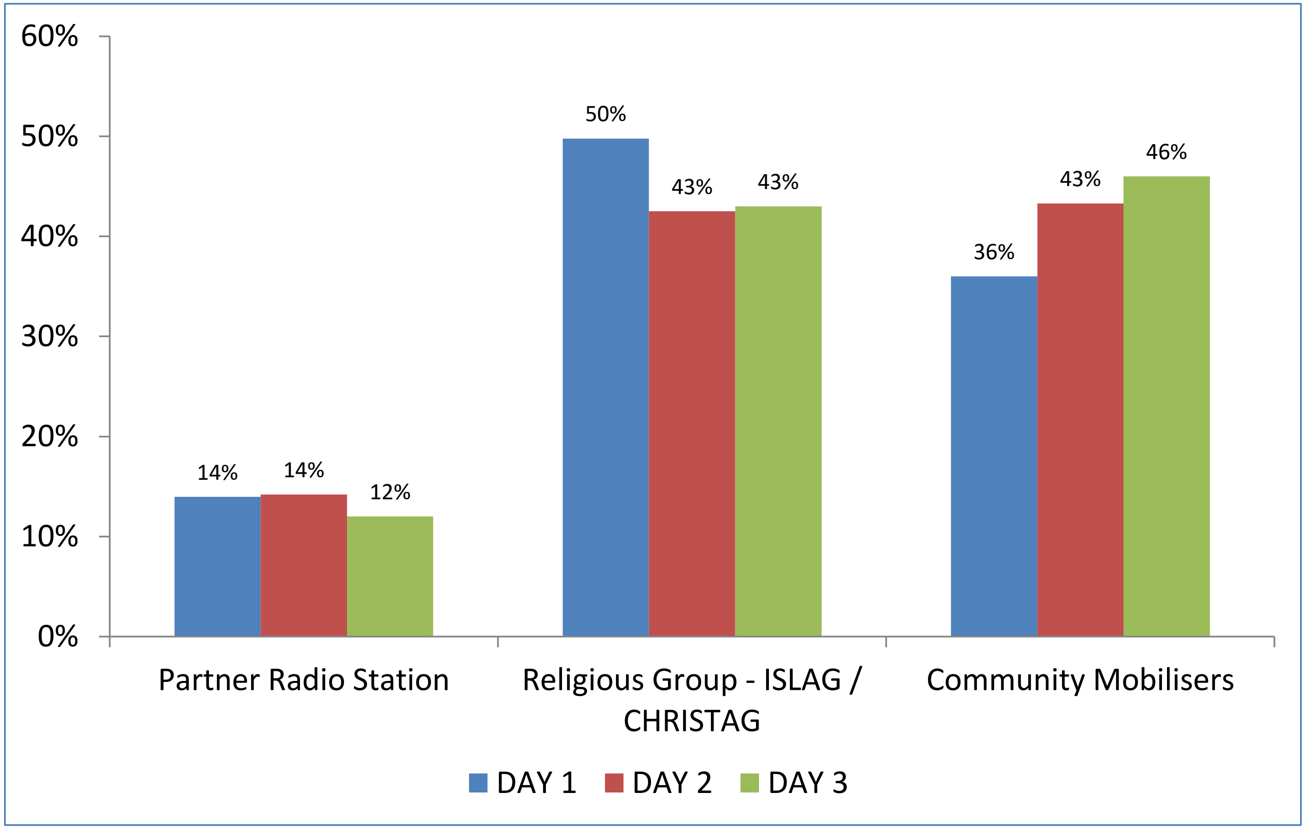

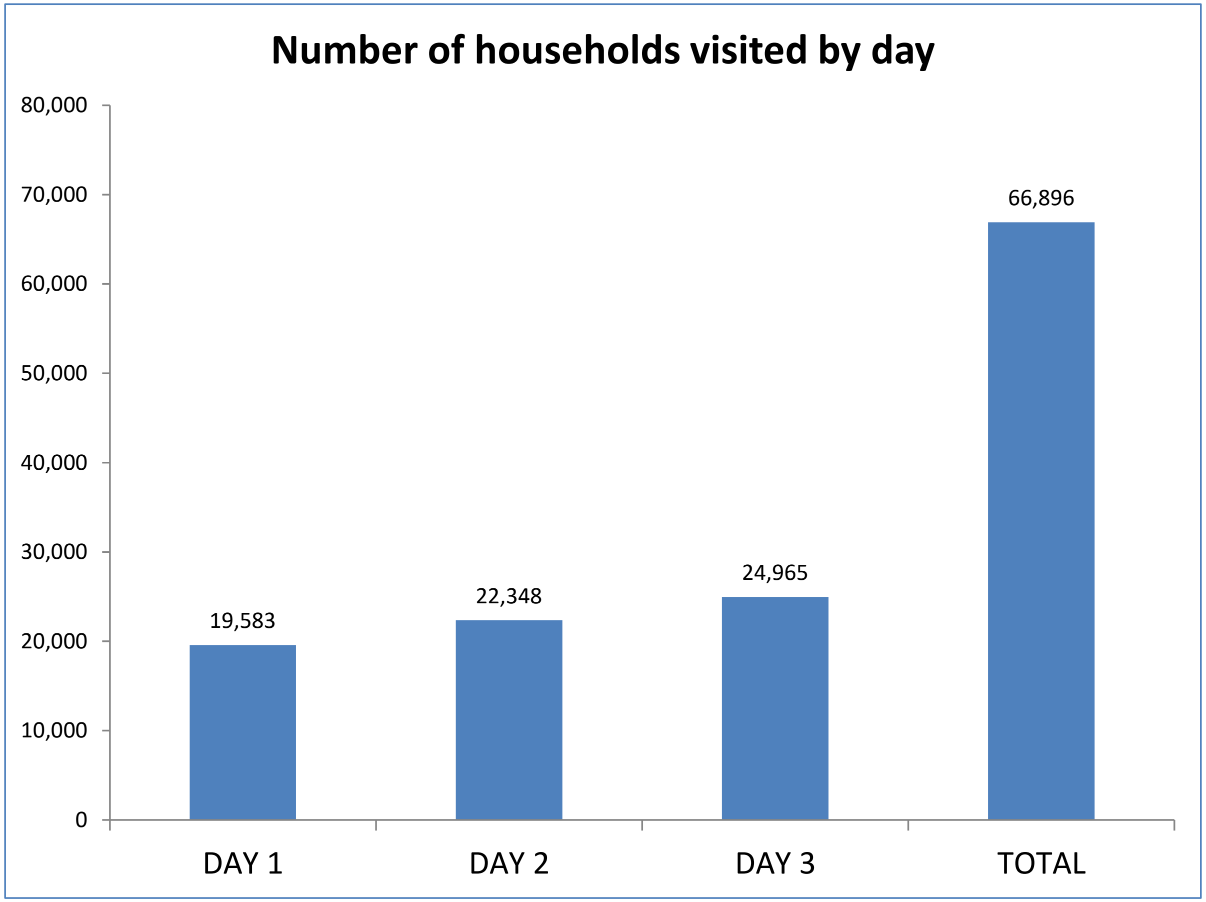
Number of households visited by day
80,000
70,000
60,000
50,000
40,000
30,000
20,000
10,000
0
19,583
22,348
24,965
66,896
DAY 1
DAY 2
DAY 3
TOTAL

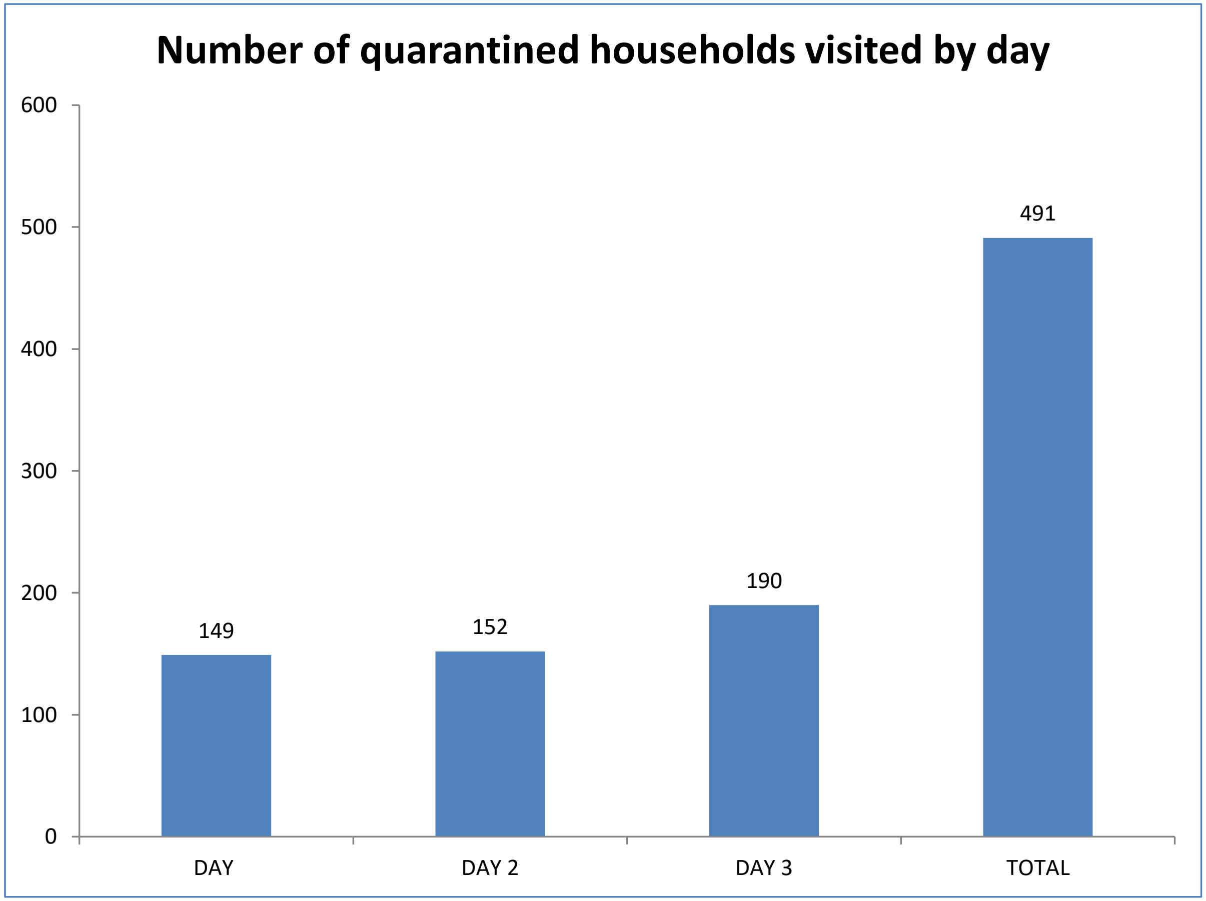
Number of quarantined households visited by day
600
500
400
300
200
100
0
149
152
190
491
DAY
DAY 2
DAY 3
TOTAL

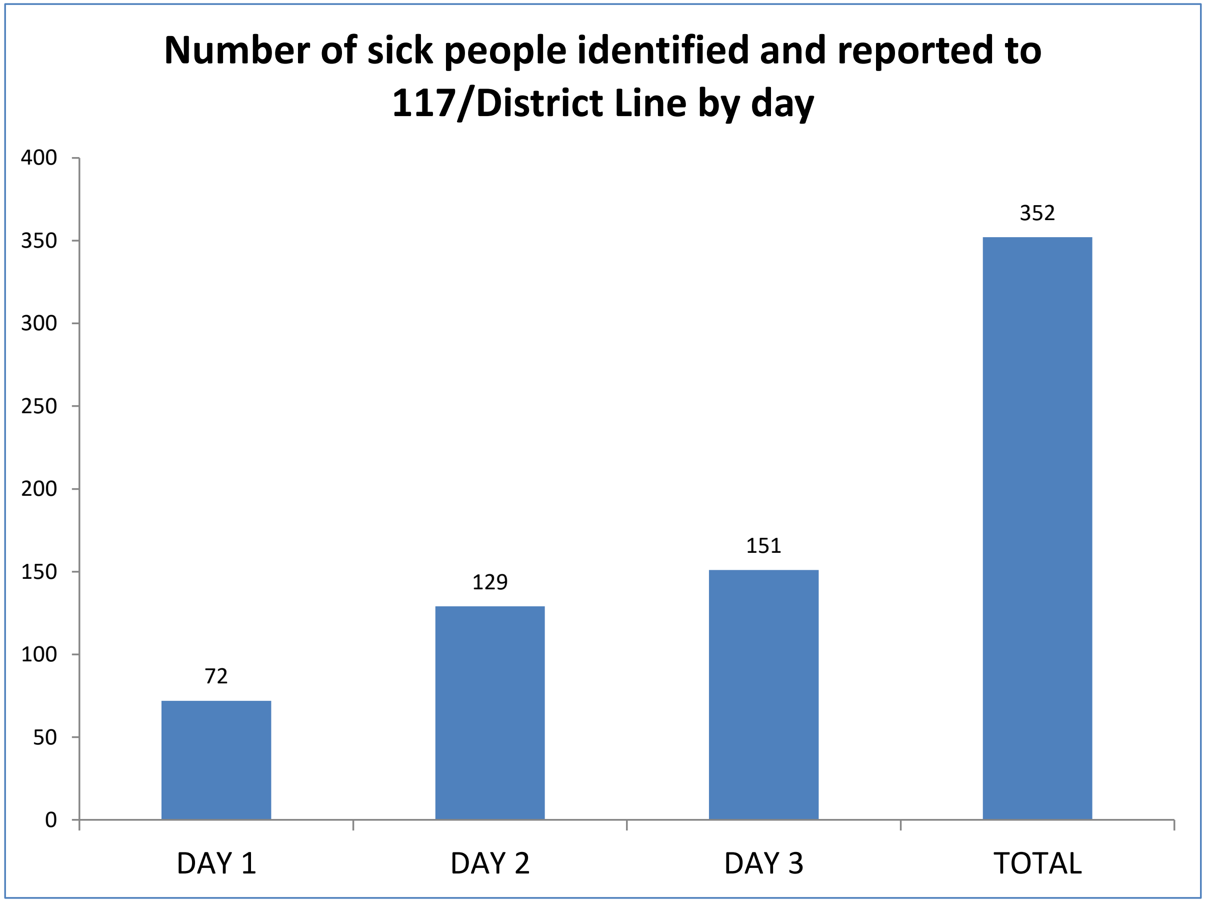
Number of sick people identified and reported to 117/District Line by day
400
350
300
250
200
150
100
50
0
72
129
151
352
DAY 1
DAY 2
DAY 3
TOTAL

BMJ Publishing Group Limited (BMJ) disclaims all liability and responsibility arising from any reliance placed on this supplemental material which has been supplied by the author(s)

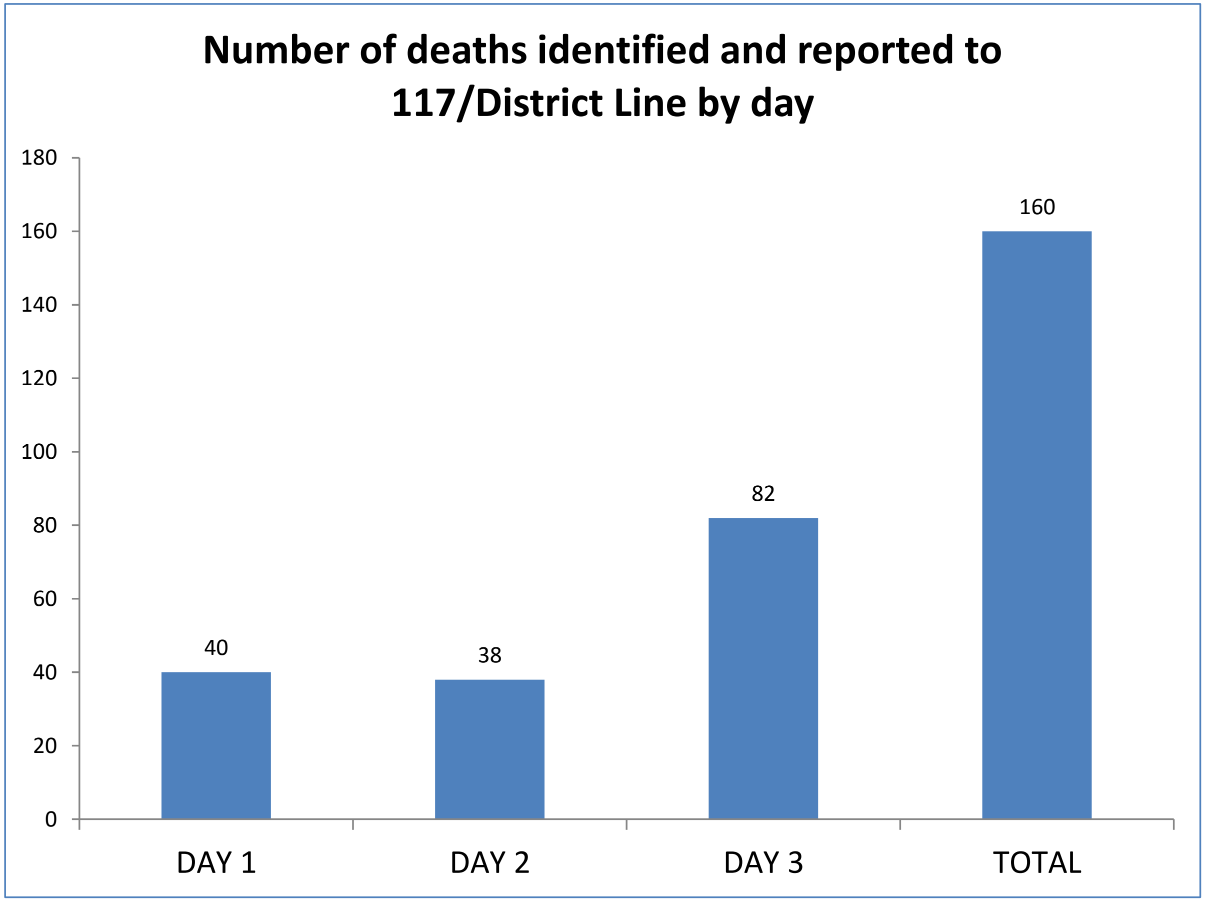

Friday Muslim “Juma” Prayer

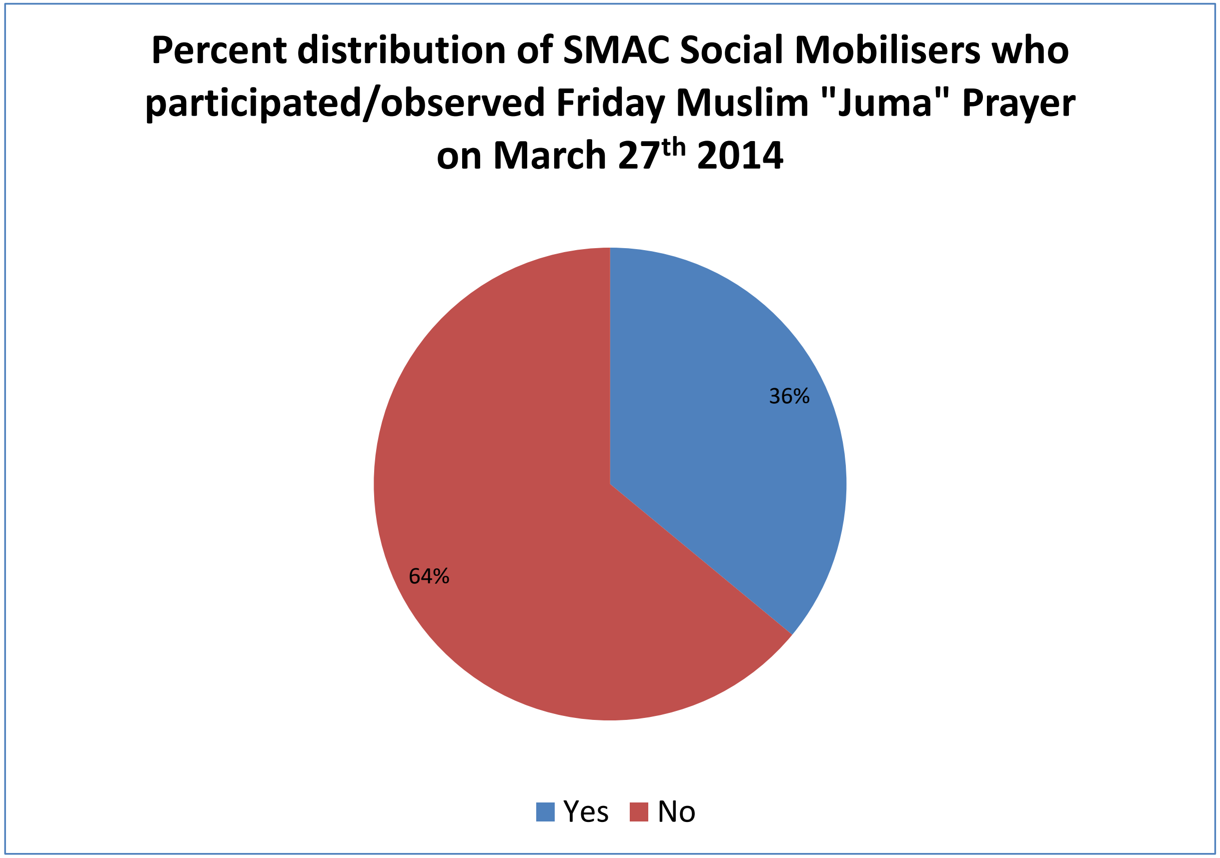

94% of Imams advised congregants to avoid touching/washing dead bodies (n=84)

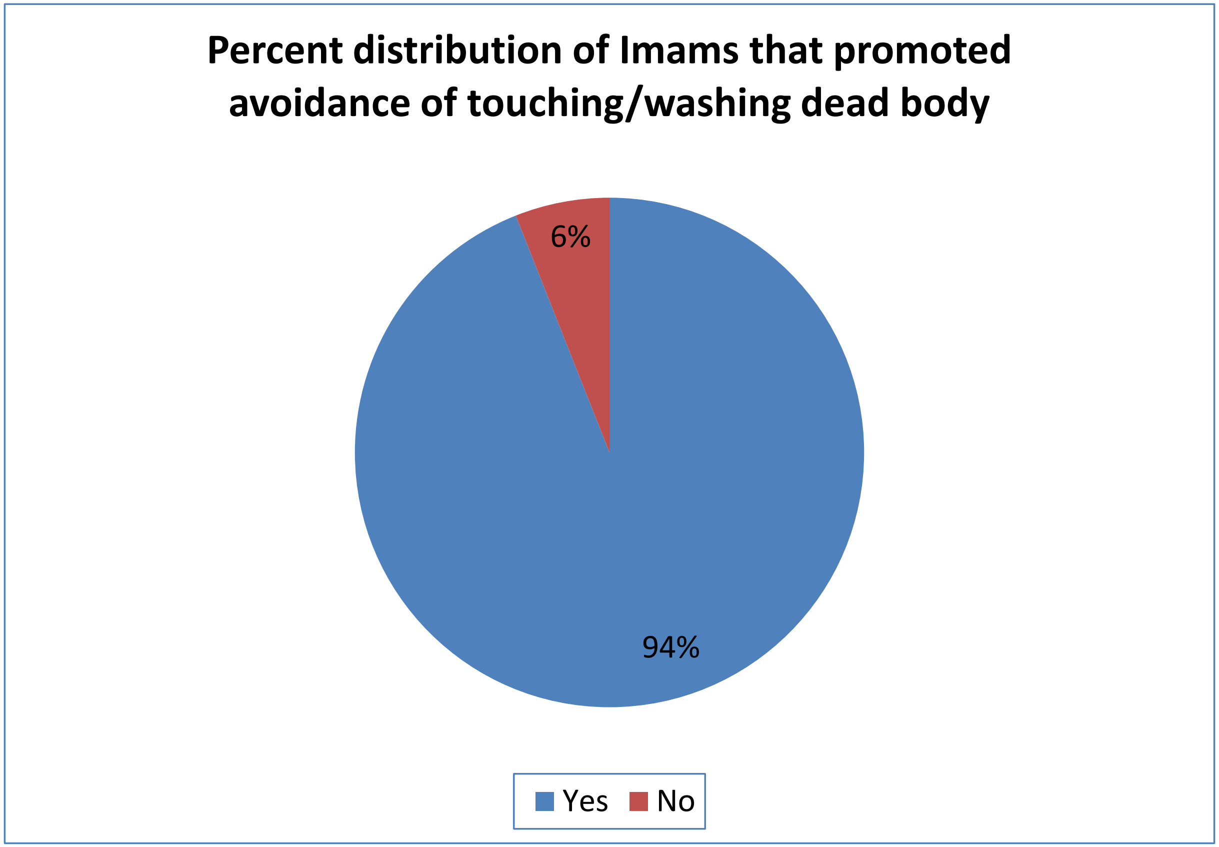

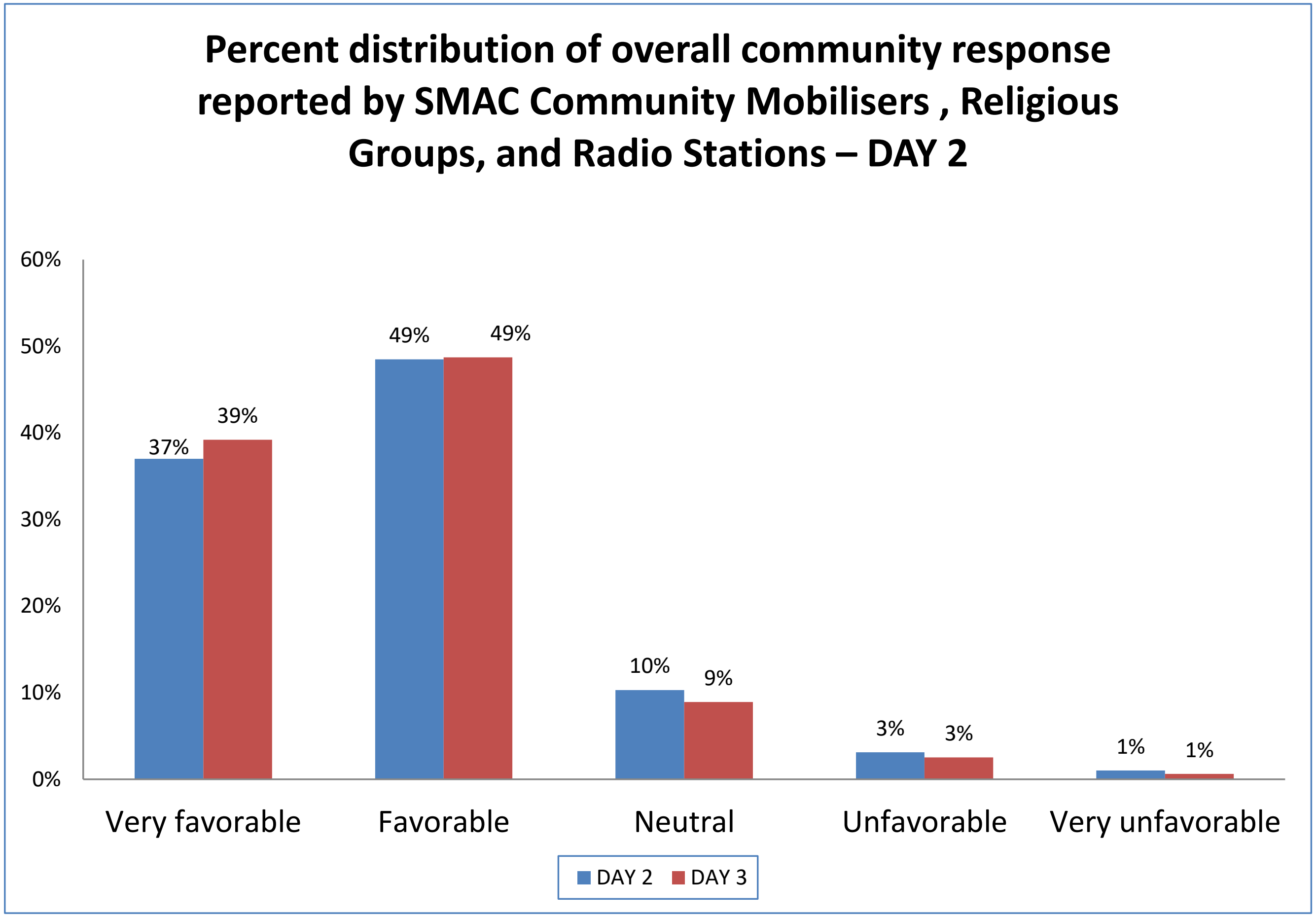
Percent distribution of overall community response reported by SMAC Community Mobilisers , Religious Groups, and Radio Stations – DAY 2
60%
50%
40%
30%
20%
10%
0%
37%
39%
49%
49%
10%
9%
3%
3%
1%
1%
Very favorable
Favorable
Neutral
Unfavorable
Very unfavorable
DAY 2
DAY 3

Qualitative Feedback

- Communities had mixed responses to the visiting teams
- Many communities were receptive to the mobilisers and the messages; expressed appreciation for the Zero Campaign
- However, in some communities – especially in Western Area – they were not well received
  - Some were refused access to the households, and accused of enjoying Ebola money
- Soap was not sufficient for all households – which created dissention in some areas
- Some quarantined homes with no food and water
- Instances of people going outside in search of good and water

Next slides:

- Illustrative quotes from SMAC Community Mobilisers, Religious Groups, and Partner Radio Stations involved in the Zero Ebola Campaign

Feedback - Western Area

"We face no challenges .The people in the community accept us and listen to what we told them."

"People were asking for food items, soap, without which they were a bit reluctant to listen the message. This was because [some] partners were given these things out . But we still pass on the message"

"Some people were aggressive because they were disgusted and tired of hearing about Ebola. They said the government has kept them at home without providing food for them."

"Well one of the challenges that I faced with the people in my community is that a truck was distributing a rice at the Portee community so my people ask me a lot of questions … why they are not supplying the Brima Lane Community … so this is one of the challenge that I face with my people

"People said that we gave them soap to wash there hands, what about the food to eat ! They raise comments about that!"

Feedback - Western Area

“Some houses we visited the people are demanding for more soap and they are saying a soap for a household of 15 is too small.”

“The challenges i faced is that am not familiar with the area. Others are shouting. Mocking of the job that..we are the one that is eating Ebola money so we don’t want Ebola to end”

“Some people are deleting the writing on the wall in order to get more soap”

“So many questions about the increase and fall of Ebola and which ways they register and gather the cases for a day. Lastly, there was no food us for 10 hours on work”

Feedback – Port Loko

“The people did not welcome us with good face at first, but after we talk to them and give reasons and they start to welcome us with good facial expression.”

“Pls note that, survivors at Petifu Portloko district Buya Romende Chiefdom complain of severe eyes problem. They therefore tendered this complain for eye medication assistance if possible.”

“They attack us about the registration WFP did to their house holds without food supply”

“They welcome and talk to me very well”

“The people are raise a challenge towards the re opening of schools”

“The soap are not enough for the community people”

Feedback - Kambia

“The people asked me to give them the soap…one each; then some abuse me [verbally].

“No challenges faced for today in my community.”

“People leave their houses for their farm huts.”

“We faced challenge's with the community but its has been solved by DHMT”

“Survivors complain of severe eye problems”

Feedback - Bombali

“People adding households to get more soap.”

“People were not ready to accept us today”

I targeted local stakeholders such as chiefs, women's leaders, youths in task force because they're in charge to ensure the process is successful. The responses I received state no case of Ebola, they are doing exactly what's told about preventing Ebola, ambulances, mobilisers, red cross journalists members of DERC are all up and about surveillancing

Feedback - Moyamba

“People were not happy to collect the soaps”

“People were saying that the soap is been poison so they will not use the soap. But they accepted it later.”

“Household members want the team to give them more than one bar soap”

“The people were asking us for food”

Feedback - Kono

“No enough soap to be distributed to dwelling/houses, complain of not giving something to eat and not enough veronical buckets for washing hands.”

“One big challenge was that food expectations, they said we are giving soap instead of rice.”

“I was faced with difficult questions but able to answer.”

“Some people were expecting supply of rice and soap.”

“Everything was okay because I worked in the place where I was born.”

BMJ Publishing Group Limited (BMJ) disclaims all liability and responsibility arising from any reliance placed on this supplemental material which has been supplied by the author(s)

THANK YOU
