# Supplementary material for: Integrated digital system for community engagement and community-based surveillance during the 2014–2016 Ebola outbreak in Sierra Leone: lessons for future health emergencies
Source: BMJ Glob Health. 2020 Dec 21;5(12):e003936. doi: 10.1136/bmjgh-2020-003936 (PMC7757454; doi:10.1136/bmjgh-2020-003936)
Supplement: Supplementary data [file bmjgh-2020-003936supp002.pdf]

BMJ Publishing Group Limited (BMJ) disclaims all liability and responsibility arising from any reliance placed on this supplemental material which has been supplied by the author(s)

Community-Based EVD Data Collection and Reporting Using Mobile Technology in Sierra Leone

Training Manual

Social Mobilisation Action Consortium

smac

Background

- SMAC national network of community groups in across the 14 districts in SL:
  - 2457 community mobilisers
  - 2000 mosques and churches / 5000+ religious leaders
  - 36 partner radio stations (national and district coverage)
- **Community Mobilisers** trained to use CLEA approach and collect community-level data (GOAL and Restless Development)
- **Mosques/Churches** trained to promote, support, and role-model EVD prevention practices (FOCUS 1000)
- **Partner Radio Stations** supported to produce high quality/interactive EVD programming (BBC Media Action)

From Paper to Smart Phone

- SMAC has been collecting social mobilization activities data and EVD alerts using paper-based instruments since November 2014.
- Recognizing the need for more rapid data collection and reporting, we are now moving to a digital data collection using Android smart phones.
- These efforts are supported by the Bill and Melinda Gates Foundation in the United States.

Five Target Districts

- The digital data collection efforts would focused on the following 5 high transmission districts during Phase I of the Gates Project:
  - Western Area
  - Kono (East)
  - Moyamba (South)
  - Kambia (North)
  - Port Loko (North)

BMJ Publishing Group Limited (BMJ) disclaims all liability and responsibility arising from any reliance placed on this supplemental material which has been supplied by the author(s)

Training Objectives

- Use KoboCollect on Android-based phones to collect and report data on SMAC activities and community-based surveillance of Ebola Virus Disease (EVD)
- Accurately submit daily surveillance data of EVD using SMAC's via SMS interaction

Training Outcome

- By the end of the training, you are expected to have acquired the technical skills required to use the SMAC Data Collection System to submit weekly and daily reports using ODK and SMS.

Training Materials

- Ensure that you have the following materials prior to the training:
  - Training Agenda
  - One-page summary
  - SMAC Phone User Registration Form
  - SMAC Phone Use Agreement
  - One phone per trainee
  - Flip charts, markers, note books, pens
  - Flash cards (for participants to write down key info)

Training Approach/Method

- The training should take on a “low-tech” hands-on approach where by participants are given sufficient opportunity to practice “doing” rather than just “listening.”
- Avoid using PowerPoint
- Move at a steady pace to ensure that no participant is left behind as this would cause disruptions down the line
- Engage all participants

BMJ Publishing Group Limited (BMJ) disclaims all liability and responsibility arising from any reliance placed on this supplemental material which has been supplied by the author(s)

Training Approach/Method

- Make an effort to identify “weak” participants, and provide them with the necessary extra support – do so in a respectful manner.
- The trainings should be educational but also entertaining and interactive. Make it an enjoyable experience.
- Provide opportunities for ice-breakers and fun activities.

Overview of SMAC Digital Data Collection System

BMJ Publishing Group Limited (BMJ) disclaims all liability and responsibility arising from any reliance placed on this supplemental material which has been supplied by the author(s)

Open Data Kit (ODK)

- ODK is an application that allows you to collect data online or offline and submit to the web. KoboCollect is based off ODK technology.

- Trained SMAC groups will submit community-based data using KoboCollect on their Android smart phones.

- Submitted data will be instantly seen by SMAC, and will inform our actions.

SMS Gateway

- SMS Gateway setup to manage mass two-way SMS
- SMAC will communicate with you via SMS on a daily basis:
  - Suspected EVD patients needing ambulance but not picked up in 24 hrs
  - deaths needing Safe Medical Burial not picked up in 24 hrs
- SMAC District Liaison Officers follow-up with DERC

BMJ Publishing Group Limited (BMJ) disclaims all liability and responsibility arising from any reliance placed on this supplemental material which has been supplied by the author(s)

Part I: Getting to Know Your Android Phone

Security

- It's important to ensure that your phone is properly secured.

- To set password for your phone:
  - Go to settings → password (the settings options may vary depending on the version of Android and the phone model)
  - In settings, ensure screen is locked after 1 min of being idle (no activity)

Power Settings

- To maximize your phone's power:
  - Reduce screen brightness to "low"
    - For most Android devices you can do this by pulling down from the top of the screen
    - Drag the indicator to at least below 50% or the middle mark
  - Ensure that the screen "timeouts" after 1 minute

Data Connectivity

- All SMAC phones are registered with Airtel.
- SMAC has a dedicated Access Point Name (APN) with Airtel.
- Phone should be configured to the SMAC APN
  - Settings → Mobile Network → Access Point Name

Data Use Restrictions

- SMAC phones can only be used to submit your weekly reports and daily SMS.
- All other use are prohibited.
- SMAC's APN with Airtel only allows access to approved sites.
- Approved data and SMS costs are pre-paid by SMAC so it's free for all our community groups!

BMJ Publishing Group Limited (BMJ) disclaims all liability and responsibility arising from any reliance placed on this supplemental material which has been supplied by the author(s)

Phone Use Policy

- By accepting your phone, you agree to comply with the terms and conditions set under SMAC Phone Use Agreement:
  - Ultimately responsible for safekeeping of the phone
  - May be held liable to replace phone if it is destroyed or stolen
  - Avoid using the phone for malicious activities

Part II: Getting to Know ODK / KoboCollect

KoboCollect

- A type of Open Data Kit (ODK) application for Android phones.
- Allows for the collection and submission of data using your phone.
- If you do not have Internet connection, KoboCollect stores the data onto your phone, and you can later submit once you regain connectivity.
- Once your data is submitted it gets uploaded to an online hosting server where SMAC can access it.

Accessing KoboCollect

- KoboCollect will be used for submitting weekly reports.
- KoboCollect has already been installed on all SMAC phones.
- You will see the KoboCollect icon on the main screen.
- You can also find it under “apps.”

Accessing KoboCollect

- Simply tap or click on the icon to get started.

- Once the app opens, it'll take you to the landing page.

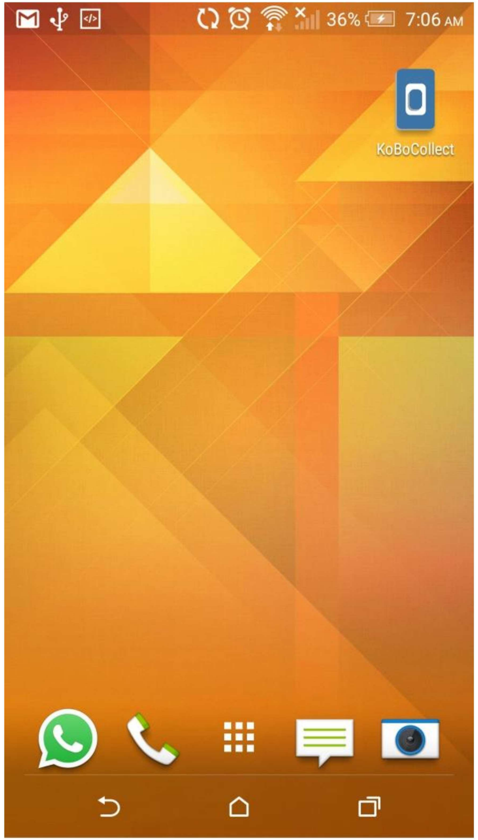

Configuring KoboCollect

- KoboCollect has been configured on all devices prior to the district roll-out,
- However, all Master Trainers should know how to configure it should issues arise out in the field.
- See next slide for screenshots illustrating the configuration.

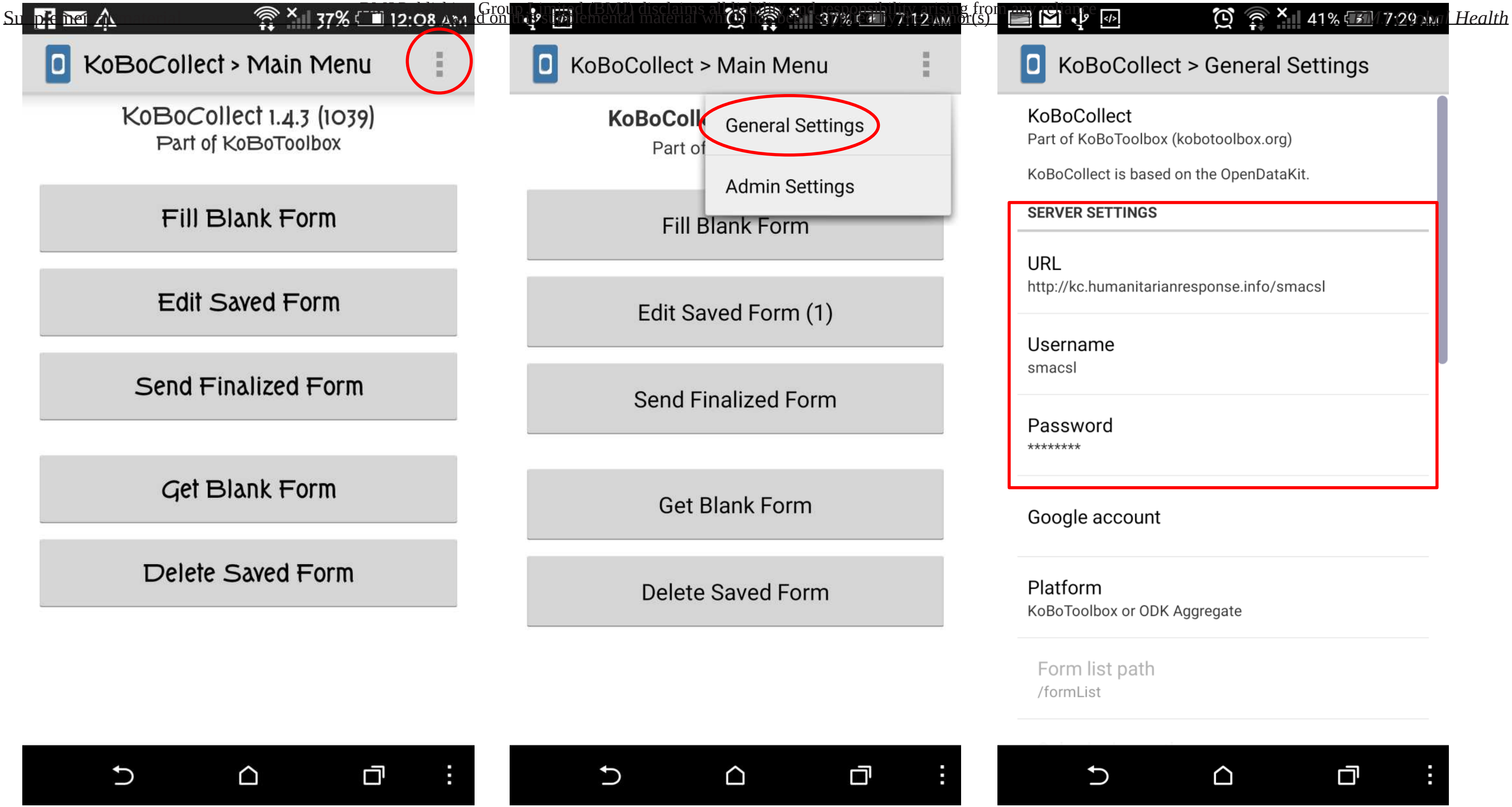

KoBoCollect > Main Menu
KoBoCollect 1.4.3 (1039)
Part of KoBoToolbox
Fill Blank Form
Edit Saved Form
Send Finalized Form
Get Blank Form
Delete Saved Form
KoBoCollect > Main Menu
General Settings
Admin Settings
Fill Blank Form
Edit Saved Form (1)
Send Finalized Form
Get Blank Form
Delete Saved Form
KoBoCollect > General Settings
KoBoCollect
Part of KoBoToolbox (kobotoolbox.org)
KoBoCollect is based on the OpenDataKit.
SERVER SETTINGS
URL
http://kc.humanitarianresponse.info/smacsl
Username
smacsl
Password
****
Google account
Platform
KoBoToolbox or ODK Aggregate
Form list path
/formList

Configuring KoboCollect

- Click on "General Settings"
- Under "Server Settings" enter the following:
  - URL: kc.humanitarianresponse.info/smacsl
  - Username: smacsl
  - Password: (already provided to Master Trainers)
  - Normal users should not access these settings
    - Even minor changes may cause the app to not work

BMJ Publishing Group Limited (BMJ) disclaims all liability and responsibility arising from any reliance placed on this supplemental material which has been supplied by the author(s)

The SMAC Form

- From the home screen, click on "Fill Blank Form" to get started

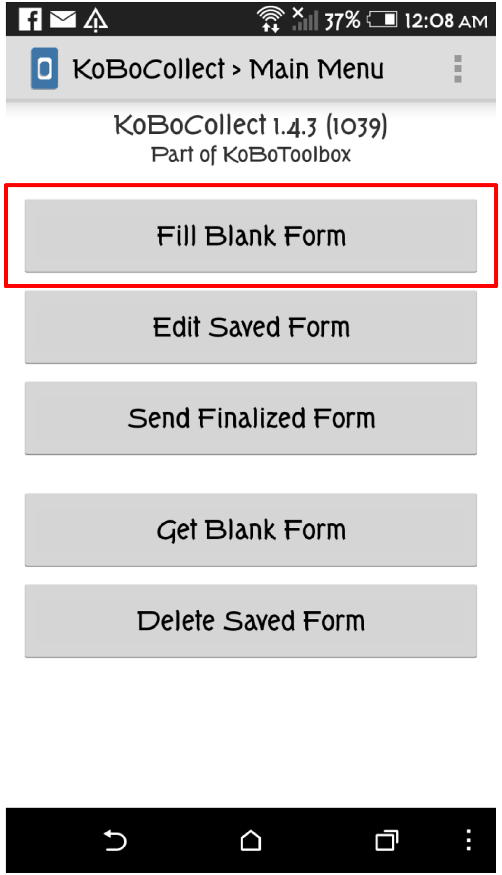

LIVE DEMO

Learning by doing

The SMAC Form

- After selecting "Fill Blank Form" select the form titled:
  - SMAC Community Data Collection Form (V4 - Final)

- Once the form has been selected, you should practice with trainees how to swipe forward and backward.

BMJ Publishing Group Limited (BMJ) disclaims all liability and responsibility arising from any reliance placed on this supplemental material which has been supplied by the author(s)

Personal Information

- Phone number (omit the leading zero)
- Full name
- Partner agency (select appropriate option)
- Partner category (select appropriate option)

Location Identification

- District
- Chiefdom / Ward (based on district selection)
- Section (if known, otherwise leave blank)
- Community (e.g. Rokupa)

At this point, Kobo will provide a summary of the info entered thus far; ensure that participants review this carefully for accuracy before proceeding to the next section

Reporting Period

- Weekly reporting period options have already been programmed for March 16th through August 2nd
- Participant should select the appropriate reporting period each week
- Beyond August 2nd they'll need to specify the "start" and "end" dates for the reporting period

Activities

- This section will ask participants to report on their respective activities undertaken during this reporting period (starting on Monday and ending on Sunday)
  - Activities will vary by SMAC partner organization
    - Community Mobilisers
    - Religious Groups
    - Radio Stations
  - Practice filling out the activities section by creating a scenario and providing dummy data

Action Plans (Mobilisers Only)

- # of action plans in place
  - This will determine the number of follow-up questions
- Action plan description
- Status of planned action point
  - Complete/achieved
  - In progress
  - Incomplete / not achieved
  - Unable to assess progress / data not available
  - New (no progress)

Action Plans (Mobilisers Only)

- Biggest change since last visit
- Most common concerns expressed by community
- Name of community rep providing updates
- Title of community rep
- Phone number of community rep

BMJ Publishing Group Limited (BMJ) disclaims all liability and responsibility arising from any reliance placed on this supplemental material which has been supplied by the author(s)

Screener Question

- After completing the "Activities" section of the form, a screener question will help determine the next set of questions to display regarding **suspected EVD cases**, **deaths**, & **survivors**
- Only applicable to mobilisers and religious groups; radio stations won't report on community surveillance data

BMJ Publishing Group Limited (BMJ) disclaims all liability and responsibility arising from any reliance placed on this supplemental material which has been supplied by the author(s)

Suspected EVD Cases (if applicable)

- Total number
- # Male 18yrs and above
- # Male 18yrs and below
- # Female 18yrs and above
- # Female 18yrs and below
- # referred to a health facility within 24hrs
- Waiting period for ambulance (within 1 day, 2 days, 3 days 4+)
- Care provided to sick while waiting for help
- Community’s experience with ambulance teams

Deaths (if applicable)

- # Male 18yrs and above
- # Female 18yrs and above
- # Male 18yrs and below
- # Female 18yrs and below
- # Buried by Burial Team
- Community's experience with Burial Team

Survivors (if applicable)

- # Male 18yrs and above
- # Female 18yrs and above
- # Male 18yrs and below
- # Female 18yrs and below
- Interviewed survivor (yes/no)
- Number of survivors interviewed
  - Will determine # of survivor surveys to pull up

Survivor Survey (if interviewed)

- For each interviewed survivor, complete the following
- Since your return, do you feel welcomed in the community?
  - Yes; no; I don't know; no response
- How would you rate your interaction with community members since your return?
  - Very good; good; not good/not bad; bad; very bad; don't know; no response

GSP Location

- For each report, the GPS location for the reported community should be submitted – its is therefore important to capture the GPS location while within the community.

- GPS coordinates can only be collected when outside and in view of the sky.

GSP Location

- Click on “Record Location”
  - Once you see “Using gaps. Accuracy is xx m”
  - Select “Record Location” – button on the right hand corner
  - You will see the following if successful
    - Latitude:….
    - Longitude:…
    - Altitude: ….
    - Accuracy:…

Photograph (if applicable)

- Emphasize to trainees that they should only submit photographs that capture something very special (e.g. high profile event) in the community.
- Submission of photo is not required, and should only be done on seldom occasions
  - Photo submissions consumes large amounts of data, and takes longer than usual to submit
- If a photo is submitted, a short description must be also be included .

BMJ Publishing Group Limited (BMJ) disclaims all liability and responsibility arising from any reliance placed on this supplemental material which has been supplied by the author(s)

Additional Info (if applicable)

- You will be asked if you have “anything else that you would like to inform SMAC about”
  - Yes /No

- Only select “yes” if you have additional information you haven’t already captured in the prior sections.

Quality Assurance and Improvement

- You will be asked several questions each week to help us improve the system.
- For instance, if you're having technical issues, you will be given the opportunity to share the specific challenge you faced.
- You'll be also be asked to rate your overall experience with the system.

Saving the Form

- Once you reach the end of the form you'll see the following →
- Ensure that the form is marked as finalized
- Click on "Save Form and Exit"

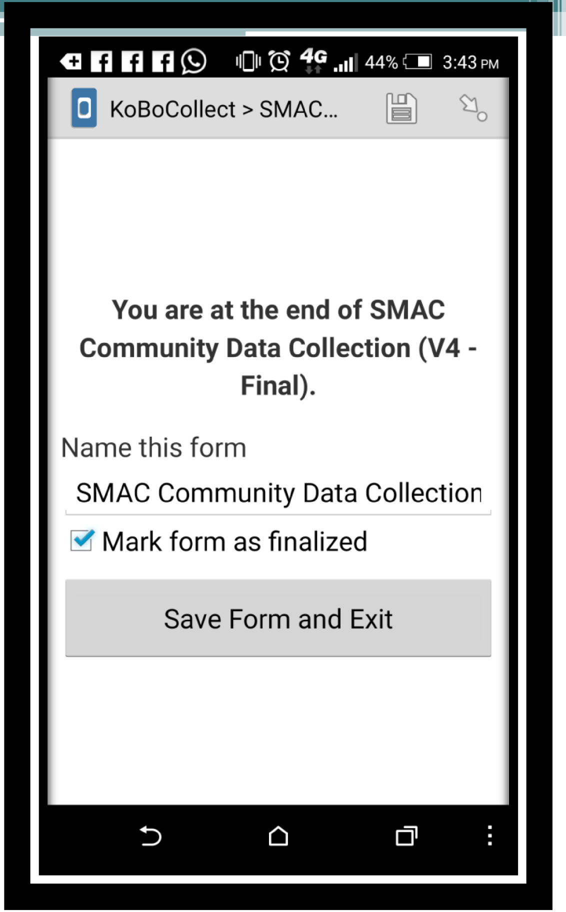

Sending the Form

- After click on "Save Form and Exit" you will taken to the landing page of Kobo
- Click on "**Send Finalized Form**"
- Click on "**Toggle All**" on the bottom panel
- Click on "**Send**"

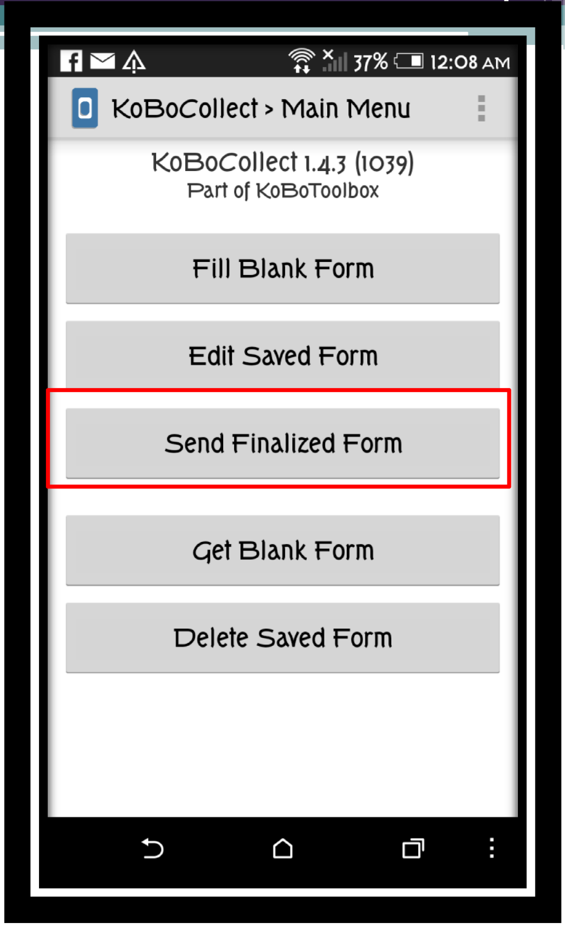

BMJ Publishing Group Limited (BMJ) disclaims all liability and responsibility arising from any reliance placed on this supplemental material which has been supplied by the author(s)

CONGRATULATIONS !!!

YOU'VE COMPLETED THE FRIST PART OF THE TRAINING ON SUBMITTIG YOUR WEEKLY REPORTS USKING KOBO COLLECT

BMJ Publishing Group Limited (BMJ) disclaims all liability and responsibility arising from any reliance placed on this supplemental material which has been supplied by the author(s)

DAY 2
Introduction to the SMAC SMS System

BMJ Publishing Group Limited (BMJ) disclaims all liability and responsibility arising from any reliance placed on this supplemental material which has been supplied by the author(s)

Introduction

- SMAC will be communicating with you daily using SMS to get information on:
  - Suspected EVD cases in your community
  - Deaths in your community
  - Issues facing quarantined homes in your community

SMAC SMS

- First, we need to register all trainees to the **SMAC SMS short code 334**

- During this segment of the training:
  - Go at a slow pace
  - Explain to trainees the type of info that they will be submitting
  - Ensure that all trainees are entering the correct information at each stage before proceeding to the next
    - Have the second trainer go around and verify registration info before sending the text at each stage

Triggering the Registration

- To start the registration:
  - Text the word "**smac**" to **334**
  - On the next page we will go through the registration process

BMJ Publishing Group Limited (BMJ) disclaims all liability and responsibility arising from any reliance placed on this supplemental material which has been supplied by the author(s)

SMS Registration -- 334

- After sending “**smac**” to **334**, you will receive the following text message:
  - “Welcome to SMAC. Let’s get you registered. What is your full name (first, middle, last)?
    - Type your full name and press send, example:
      - “Alusine Momoh Kamara”

Registration - Gender

- You will receive the following text:
  - What is your gender? (1) Male (2) Female
    - Reply with the number "1" if you're a male
    - Reply with the number "2" if you're a female

Registration - Age

- **You will receive the following text:**
  - What is your age in numbers?
    - Reply back with your age (years); example “36”

Registration – Partner Agency

- **You will receive the following text:**
  - Which SMAC partner agency are you working with? (1) BBC Media (2) FOCUS 1000 (3) GOAL (4) Restless Development
    - Reply back with the number “1” “2” “3” or “4” only

Registration – Partner Category

- You will receive the following text:
  - Thanks. Reply with (1) for Mobiliser (2) for Religious Group (3) for Radio Station
    - Reply back with the number “1” “2” or “3” only

Registration – District

- **You will receive the following text:**
  - Which district are you in? Reply (1) Western Area (2) Bombali (3) Kambia (4) Koinadugu (5) Port Loko (6) Tonkolili (7) Kailahun (8) Kenema (9) Kono (10) Bo (11) Bonthe (12) Moyamba (13) Pujehun

    - Enter the number 1 through 13 only depending on the district where you reside

    - Other values will be rejected by the system, and you will be prompted to re-enter a correct number b/w 1 and 13

Registration – END

- **If your registration is successful, you will receive the following text message:**
  - “Thanks! You are now registered with SMAC. We will be in touch”

Sample Registration

On the next page we will show you sample registration screenshots for the following user:

- Name: Alusine Momoh Kamara
- Gender: Male
- Age: 35 years
- Partner Agency: Restless Development
- Partner Category: Community Mobiliser
- District: Kambia

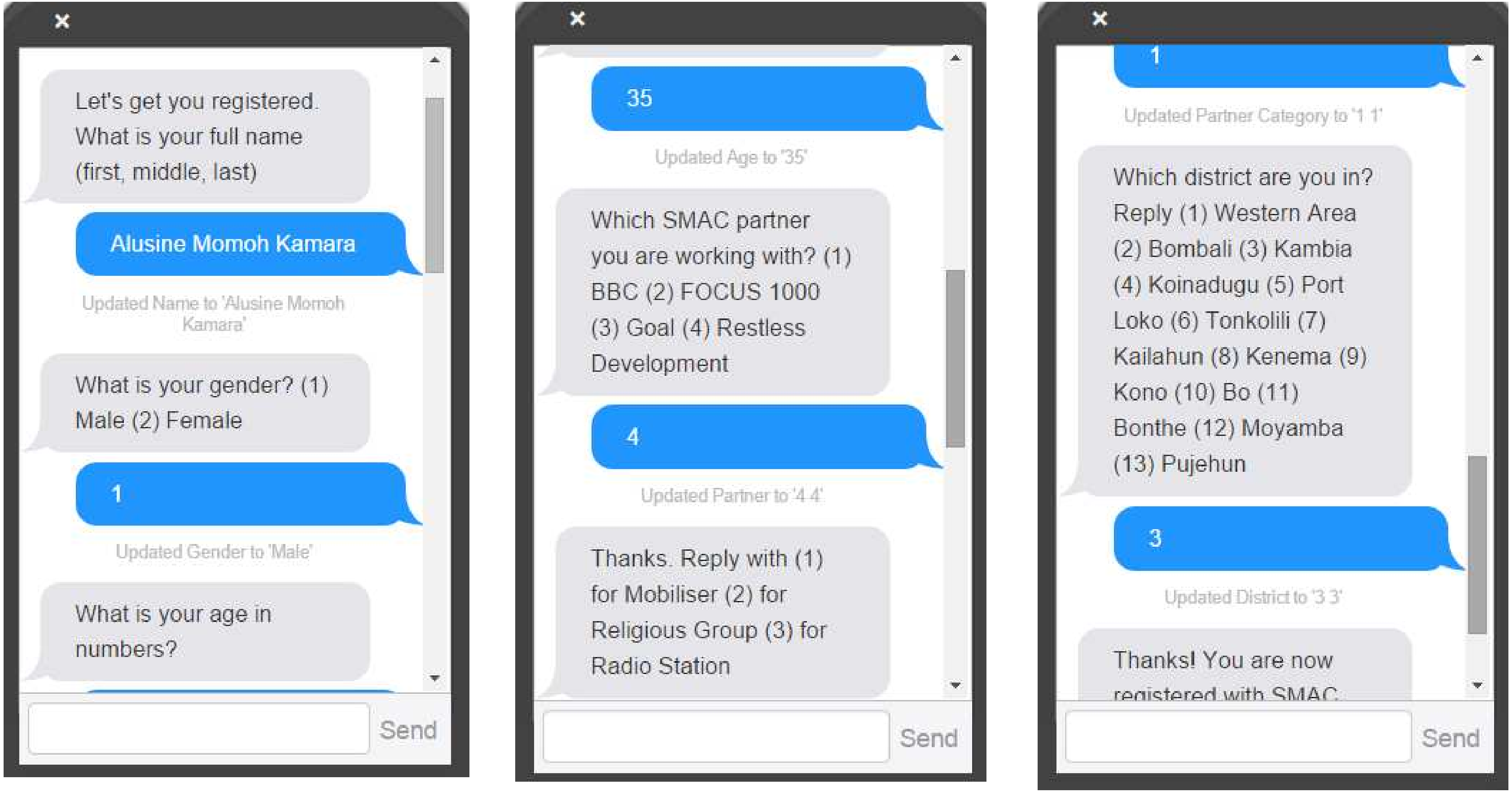
Let's get you registered. What is your full name (first, middle, last)
Alusine Momoh Kamara
Updated Name to 'Alusine Momoh Kamara'
What is your gender? (1) Male (2) Female
1
Updated Gender to 'Male'
What is your age in numbers?
Send
35
Updated Age to '35'
Which SMAC partner you are working with? (1) BBC (2) FOCUS 1000 (3) Goal (4) Restless Development
4
Updated Partner to '4 4'
Thanks. Reply with (1) for Mobiliser (2) for Religious Group (3) for Radio Station
Send
1
Updated Partner Category to '1 1'
Which district are you in? Reply (1) Western Area (2) Bombali (3) Kambia (4) Koinadugu (5) Port Loko (6) Tonkolili (7) Kailahun (8) Kenema (9) Kono (10) Bo (11) Bonthe (12) Moyamba (13) Pujehun
3
Updated District to '3 3'
Thanks! You are now
Send

Registration

- Note
  - **Do not** conduct a sample registration with trainees.
  - **All registrations must be "real"** with actual information for the respective phone user.
  - Once registered, the phone number will always be associated with the respective user.
  - Mistakes should be avoided – however, if the registration info is entered incorrectly please stop at that stage and have the user re-start the registration by sending "**smac**" to **334**.
  - Inform FOCUS 1000 of these situations immediately after the training.

SMS Alerts

- If you encounter / observe the following events in your community you should send an alert to SMAC:
  - Suspected EVD case
  - Death
  - Quarantined homes facing challenges with basic supplies (e.g. food)
  - Violence towards survivors
  - Orphaned children

SMS Alerts – Suspected EVD Case

- To report a suspected case text the word "**sick**" to **334**
- You will then be asked for the following:
  - Have the authorities been notified?
    - Who was notified?
      - How long since notified?
- Someone from SMAC will call you to follow-up

SMS Alerts – Death

- To report a suspected case text the word “**death**” to **334**

- You will then be asked for the following:
  - Have the authorities been notified?
    - Who was notified?
      - How long since notified?
- Someone from SMAC will call you to follow-up

SMS Alerts – Quarantined Home

- To report a suspected case text the word "**help**" to **334**
- You will then be asked for the following:
  - What is the type of issue facing the quarantined home?
    - Who has the issue been reported to?
      - How long since reported?
- Someone from SMAC will call you to follow-up

SMS Alerts – Quarantined Home

- To report issues facing quarantined homes, text the word "**help**" to **334**

- You will then be asked for the following:
  - What is the type of issue facing the quarantined home?
    - Who has the issue been reported to?
      - How long since reported?

- Someone from SMAC will call you to follow-up

SMS Alerts – Survivors

- To report violence or discrimination towards Ebola Survivors text the word “**hero**” to **334**

- You will then be asked for the following:
  - Type of violence?
  - Perpetrator?
  - Who has the issue been reported to?
  - How long since reported?

- Someone from SMAC will call you to follow-up

SMS Alerts – Orphans

- To report an identified Ebola Orphan text the word “**child**” to **334**
- You will then be asked for the following:
  - Age (if known)?
  - Gender?
  - Current living situation?
    - Relatives, community members, street child
- Someone from SMAC will call you to follow-up

BMJ Publishing Group Limited (BMJ) disclaims all liability and responsibility arising from any reliance placed on this supplemental material which has been supplied by the author(s)

Daily SMS

- At 5pm each day you will receive a text message from SMAC to check on any of the folloiwing that you haven't already reported via SMS:
  - Suspected EVD cases in your community
  - Deaths in your community
- If you have no suspected cases or deaths, you should still reply and confirm that this is in fact the case.

Initial Daily SMS

- You will receive the following message daily around 5pm in the evening

- Are you aware of a suspected EVD case or death in your community that you haven't already informed SMAC about thru SMS?
  - Reply with "Yes" or "No"
    - If yes, we will send you follow-up text messages

Daily SMS – Follow-up

You will then receive a follow-up text asking:

- "Reply with (1) for suspected case (2) for dead body"
- The system will only accept "1" or "2" – all other values will be rejected, and you will be asked to re-enter a valid response

Suspected case: 1
Death: 2

Daily SMS – Follow-up

You will then be asked the following:

- "Have the authorities been notified? Please reply with Yes or No."

If yes, you will then be asked:

- Which authority has been notified? (1) for 117 (2) for District Line (3) for Chief (4) for Other

SMS – Suspected EVD Case

Assuming you entered "1" you will then be asked the following:

- "Have the authorities been notified? Please reply with Yes or No."

If yes, you will then be asked:

- Which authority has been notified? (1) for 117 (2) for District Line (3) for Chief (4) for Other

BMJ Publishing Group Limited (BMJ) disclaims all liability and responsibility arising from any reliance placed on this supplemental material which has been supplied by the author(s)

SMS – Suspected EVD Case

You will then be asked:

- "How long has it been since the authorities were notified? Reply (1) one day (2) two days (3) days or more"

This will be followed by:

- "Thank you. Are you aware of another suspected EVD case or death in the community? Reply with yes or no"

BMJ Publishing Group Limited (BMJ) disclaims all liability and responsibility arising from any reliance placed on this supplemental material which has been supplied by the author(s)

SMS – Death

- “Reply with (1) for suspected case (2) for dead body”

You will reply with the number “2”

SMS – Death

You will then be asked the following:

- “Have the authorities been notified? Please reply with Yes or No.”

If yes, you will then be asked:

- Which authority has been notified? (1) for 117 (2) for District Line (3) for Chief (4) for Other

SMS – Death

You will then be asked the following:

- “Have the authorities been notified? Please reply with Yes or No.”

If yes, you will then be asked:

- Which authority has been notified? (1) for 117 (2) for District Line (3) for Chief (4) for Other

SMS – Death

You will then be asked:

- “How long has it been since the authorities were notified? Reply (1) one day (2) two days (3) days or more”

This will be followed by:

- “Thank you. Are you aware of another suspected EVD case or death in the community? Reply with yes or no.”

Multiple suspected cases or deaths

- You will need to submit a separate report for each suspected case or death in your community.
- At the end of every report, you will always be given asked if there are other suspected cases or deaths that you'd like to report.
- The process will continue until all suspected cases and deaths in your community have been reported.

Reporting Guidelines

- You must always first report cases to the appropriate authorities before reporting to SMAC – **our system does not replace the existing systems of 117 or district lines**.

- Depending on the locality, it may be that you must first alert the chief before reporting to 117 or district lines – in such instances, we ask that you follow these bye-laws.

Reporting Guidelines

- **Before reporting to SMAC**, please make sure that you have as much information about the suspected case or death by talking to family and community members.

- Key information that we will ask for you are outlined on the next page.

Key Follow-up Information

- Someone from SMAC will call you to get additional information on the reported subject(s) including:
  - Name
  - Gender
  - Age
  - Location (District, Chiefdom, Community, Street, etc.)
  - Date / time authorities were notified
  - Any other helpful background information

- The more detail you can provide the better!

BMJ Publishing Group Limited (BMJ) disclaims all liability and responsibility arising from any reliance placed on this supplemental material which has been supplied by the author(s)

District Liaison Officers (DLOs)

- The DLOs will be tasked with following-up on the reported suspected cases, deaths, and issues facing quarantined homes.

- In addition, the DLOs and the SMAC Team will work with you and others to conduct tailored community engagement on the emerging issues – including providing feedback to the community

Other SMS

- From time to time we may send you text messages about other issues in your community. We expect that you will:
  - Read all text messages sent from 334
  - Respond accordingly to the best of your knowledge
    - Usually with a number: 1, 2, 3, 4, 5…
    - Or sometimes with a "yes" or "no"

BMJ Publishing Group Limited (BMJ) disclaims all liability and responsibility arising from any reliance placed on this supplemental material which has been supplied by the author(s)

CONGRATULATIONS AGAIN!!!

YOU HAVE NOW REACHED THE END OF THE TRAINING.

WE LOOK FORWARD TO GETTING VALUABLE INFORMATION FROM YOU AND YOUR COMMUNITY.

YOU ARE PLAYING AN IMPORTANT ROLE IN THE FIGHT AGAINST EBOLA IN SIERRA LEONE.

THANK YOU FOR YOUR COMMITMENT AND HARD WORK!

BMJ Publishing Group Limited (BMJ) disclaims all liability and responsibility arising from any reliance placed on this supplemental material which has been supplied by the author(s)

Authored by: FOCUS 1000 (www.focus1000.org)
